# An Unusual Case of Biatrial Myxoma in a Young Female

**DOI:** 10.1155/2016/3545480

**Published:** 2016-01-03

**Authors:** Vikrant Vijan, Anjith Vupputuri, Rajiv Chandrasekharan Nair

**Affiliations:** Amrita Institute of Medical Sciences (AIMS), Amrita Vishwa Vidyapeetham University, Kochi 682041, India

## Abstract

Myxoma, a rare type of intracardiac tumor, forms a very small percentage of the cardiac cases. Reports of biatrial myxoma are rarer, with cases of single tumor reaching both atria being more common. Here, we present an unusual case of two independently growing atrial myxoma in a 29-year-old female. We emphasize that early recognition of symptoms, confirmation of diagnosis by transesophageal echocardiography, and prompt surgical excision remain vital in the management of such patients. The patient in the present case was managed successfully with no evidences of recurrence at the last follow-up.

## 1. Introduction

Myxoma is a rare type of intracardiac tumor with an incidence of 0.0017% among the general population [1]. The left atrium is the most common site of origin of cardiac myxoma (75–80%), followed by right atrium (10–20%). Multiple myxoma represent 5% of all cardiac myxoma, of which half (2.5%) are of bilateral origin [1, 2]. According to the available literature, only a few cases of biatrial myxoma have been reported so far, and they generally describe a single tumor reaching both atria [3]. Herein, we present a very rare case of two independently growing atrial myxoma in a young female.

## 2. Case Report

**Figure 1:**
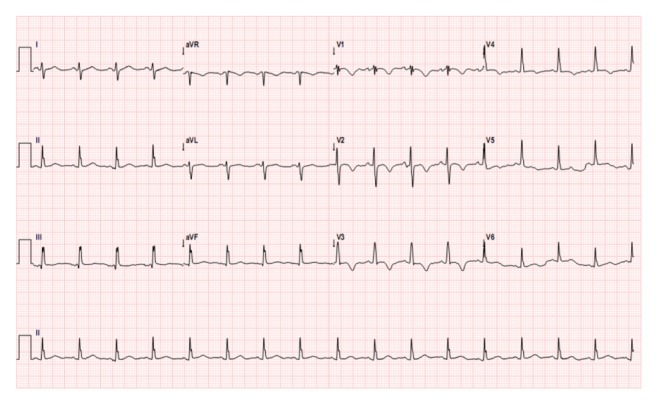
Electrocardiogram showing T-wave inversion in leads III and V1–4.

**Figure 2:**
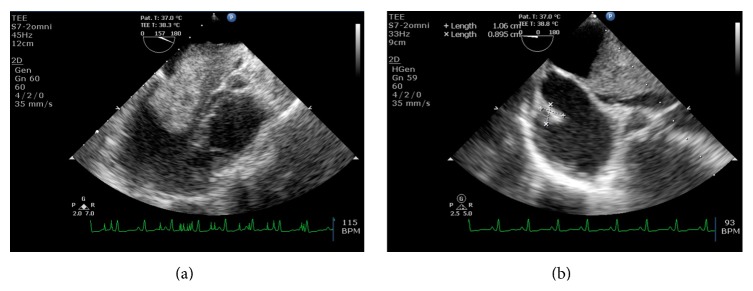
2D transesophageal echocardiogram revealed (a) large left atrial mass of 6.6 × 3.1 cm size and (b) right atrial mass of 1 × 1 cm size.

**Figure 3:**
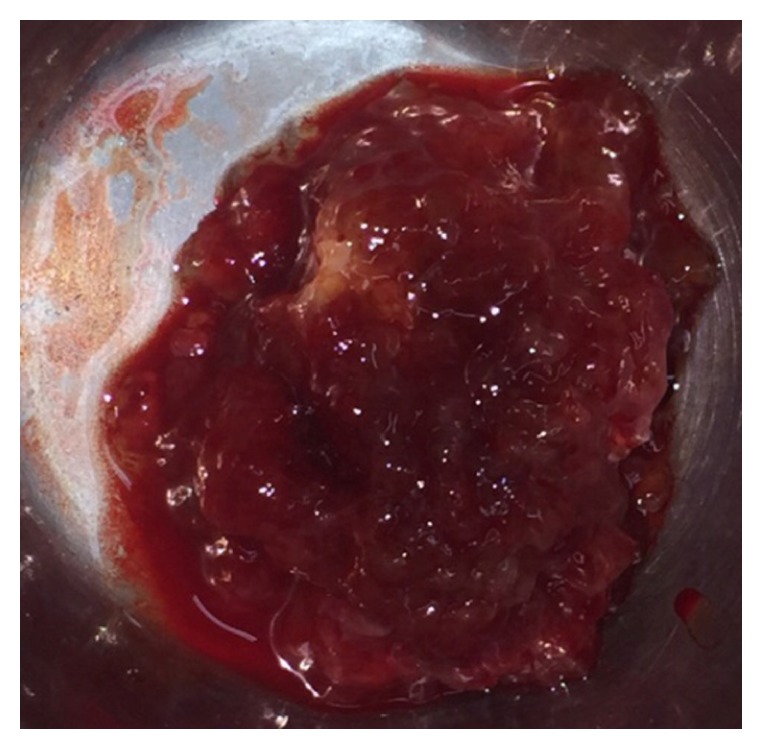
Macroscopic view of the surgically excised atrial mass.

A 29-year-old female presented to our institute with class III dyspnea and a history of cough for the past 1 year. She denied any past history of cardiac illness. Cardiac auscultation revealed a middiastolic murmur across mitral valve, followed by a tumor plop. Patient's electrocardiogram (Figure 1) showed normal sinus rhythm; right axis deviation; and T-wave inversion in leads III and V1–4. Her pulse rate was 110 beats/min. Subsequently, transthoracic echocardiography in the emergency room revealed left atrium enlargement with an evidence of left atrial mass prolapsing through the mitral valve during diastole. The enlarged left atrial mass was probably attached to the interatrial septum. In addition, there were signs of mild eccentric mitral regurgitation and moderate tricuspid regurgitation. Left ventricle was of normal size with intact ventricular septum. The patient was further analyzed by transesophageal echocardiography. It revealed large left atrial mass attached to the interatrial septum (size: 6.6 × 3.1 cm; Figure 2(a)), with mobility into and out of the left ventricular inflow. Of significance, another mass was also evident in the right atrium (size: 1 × 1 cm; Figure 2(b)). Mitral valve appeared structurally normal. Notably, the right atrial mass was a separate entity and was not attached to interatrial septum. Immediate operation was indicated and the patient was scheduled for the surgical excision of these masses with cardiopulmonary bypass through right atrial approach. Both atrial masses were removed successfully along with the interatrial septum. Further, the interatrial septum was replaced with a pericardial patch. Upon histopathological examination, atrial masses were diagnosed as cardiac myxomas based on their suggestive features. The left atrial myxoma measured 6.5 × 5 × 3 cm, while right atrial myxoma measured 1.2 × 0.8 × 0.2 cm (Figure 3). According to our examinations and laboratory evaluation, hemorrhage was established as the most probable cause for the formation of cystic lesions seen in the masses. Simultaneous screening of patient's family members revealed neither a family history nor features suggestive of cardiac myxoma.

Transthoracic echocardiogram performed at the 6-month follow-up in our patient revealed normal cardiac chambers, improved left ventricular function, and no evidence of residual myxoma or myxoma recurrence. The patient was kept on a yearly follow-up and she has remained symptom-free for about 1.5 years after the procedure.

## 3. Discussion

Myxoma is the most prevalent primary tumor of the heart. Despite being benign, it may lead to embolic events or even sudden death. Early recognition, diagnosis, and prompt treatment remain vital to prevent life-threatening events in such patients [2].

In general, patients with cardiac myxoma present with symptoms of hemodynamic obstruction, embolization, or constitutional changes. Transesophageal echocardiography is widely accepted as the ideal diagnostic tool for cardiac myxoma. It confirms the location and extension of the tumor as well as the site of attachment of the tumor. Once the diagnosis is established, immediate surgical treatment is indicated in all patients to avoid further tumor embolism and valve obstruction. Prognosis is excellent after surgical excision. After surgery, regular follow-up with serial echocardiography is also very important to detect recurrence [1, 2].

The present case is rare and particularly interesting because there were two different tumors, coexisting in each atrium. Initially, the diagnosis was not clearly established on transthoracic echocardiographic examination and the second tumor in the right atrium was missed. On the other hand, transesophageal echocardiography was effective at detecting both atrial tumors and was particularly helpful in identifying the site of tumor attachment and characteristics of the mass. Therefore, we recommend the use of transesophageal echocardiography as an ideal noninvasive diagnostic modality in cases of cardiac myxoma. It is estimated that the average growth rate of left atrial myxoma ranges from 1.8 to 5.7 cm/year [4]. Accordingly, the patient in the present case seems to have cardiac myxoma for more than one year as the dimension of left atrial mass in our case was 6.6 × 3.1 cm. This was reflected in patients' symptoms, that is, complaints of class III dyspnea for 1 year at the time of presentation. Other important features in our case include presentation of biatrial myxoma at relatively young age and absence of family history of myxoma. Available literature indicates that most of the cardiac myxomas are found in middle-age females and a small percentage of primary cardiac tumors may be familial [1].

Although prompt excision with high-risk cardiopulmonary bypass is the only acceptable mode of treatment [1], we believe that cardiac myxomas can be excised successfully with a low rate of mortality and morbidity. The patient in the present case was successfully operated on and is not having any recurrence to date.

## References

[1] Azari A., Moravvej Z., Chamanian S., Bigdelu L. (2015). An unusual biatrial cardiac myxoma in a young patient. *The Korean Journal of Thoracic and Cardiovascular Surgery*.

[2] O'Brien-Connors M. (2004). Biatrial myxoma: rare incidence in cardiac surgery. *Canadian Journal of Cardiovascular Nursing*.

[3] Schneider S., Dell'Aquila A., Martens S., Rukosujew A. (2014). Biatrial recurrence of two independently growing cardiac myxoma in a patient with multiple tumor disease. *The Thoracic and Cardiovascular Surgeon Reports*.

[4] Namura O., Saitoh M., Moro H. (2007). A case of biatrial multiple myxomas with glandular structure. *Annals of Thoracic and Cardiovascular Surgery*.

